# Recent advances in organocatalytic asymmetric aza-Michael reactions of amines and amides

**DOI:** 10.3762/bjoc.17.173

**Published:** 2021-10-18

**Authors:** Pratibha Sharma, Raakhi Gupta, Raj Kumar Bansal

**Affiliations:** 1 Department of Chemistry, The IIS (deemed to be University), Jaipur 302 020, India

**Keywords:** asymmetric aza-Michael reaction, covalent bonding catalysis, nitrogen heterocycles, non-covalent bonding catalysis, organocatalysis

## Abstract

Nitrogen-containing scaffolds are ubiquitous in nature and constitute an important class of building blocks in organic synthesis. The asymmetric aza-Michael reaction (aza-MR) alone or in tandem with other organic reaction(s) is an important synthetic tool to form new C–N bond(s) leading to developing new libraries of diverse types of bioactive nitrogen compounds. The synthesis and application of a variety of organocatalysts for accomplishing highly useful organic syntheses without causing environmental pollution in compliance with ‘Green Chemistry” has been a landmark development in the recent past. Application of many of these organocatalysts has been extended to asymmetric aza-MR during the last two decades. The present article overviews the literature published during the last 10 years concerning the asymmetric aza-MR of amines and amides catalysed by organocatalysts. Both types of the organocatalysts, i.e., those acting through non-covalent interactions and those working through covalent bond formation have been applied for the asymmetric aza-MR. Thus, the review includes the examples wherein cinchona alkaloids, squaramides, chiral amines, phase-transfer catalysts and chiral bifunctional thioureas have been used, which activate the substrates through hydrogen bond formation. Most of these reactions are accompanied by high yields and enantiomeric excesses. On the other hand, N-heterocyclic carbenes and chiral pyrrolidine derivatives acting through covalent bond formation such as the iminium ions with the substrates have also been included. Wherever possible, a comparison has been made between the efficacies of various organocatalysts in asymmetric aza-MR.

## Introduction

The Michael reaction though discovered about 135 years ago [1–2] continues to attract attention of the chemists owing to its potential of making a vast variety of organic compounds particularly of pharmacological importance accessible. Over the years, its many versions known as aza-Michael, thio-Michael, oxa-Michael, phospha-Michael, etc. have been developed and well exploited for their synthetic applications [3–7]. The reaction involving a nitrogen-based nucleophile as the Michael donor is known as the aza-Michael reaction (aza-MR). In view of its ability to introduce a nitrogen-containing functionality at the β-position of an activated alkenyl- or alkynyl-substrate, over the years, it has developed as an important synthetic strategy for the preparation of a large variety of β-amino carbonyl and similar motifs which are present in many bioactive natural products [8–9], antibiotics [10–12] and chiral auxiliaries [13–15]. However, the reaction of many nitrogen-nucleophiles, such as aromatic amines, amides, imides, etc. require the use of an appropriate catalyst to undergo a Michael addition with a suitable acceptor. In view of this, chemists endeavoured to develop different types of catalysts, particularly the chiral catalysts to accomplish asymmetric aza-MRs. The development of metal-free small organic molecules as catalysts has been a landmark advancement in organic synthesis in the recent past [16]. MacMillan and co-workers for the first time in the year 2000 termed these catalysts as ‘Organocatalysts’ [17]. It was followed by intense activity and phenomenal rise in the number of publications in this field. These organocatalysts have been found compatible with many aspects of ‘Green Chemistry’ on the one hand, and highly selective in many organic syntheses on the other hand [17]. It has an added advantage that a large number of enantiomerically pure organocatalysts can be accessed from the chiral pool. Both types of organocatalysts, namely those acting through non-covalent bonding as well as those working by making covalent bonding have been employed for accomplishing asymmetric aza-MRs.

There are several review articles available on organocatalytic asymmetric aza-MRs, each highlighting a certain aspect of the reaction. While Sánchez-Roselló et al. [18] classified these reactions on the basis of the nature of the substrates, Nayak et al. [19] and Bhanja et al. [20] focused on the stereoselective synthesis of nitrogen heterocycles via Michael cascade reactions. Recently, Vinogradov et al. [21] reviewed the synthesis of pharmacology-relevant nitrogen heterocycles via stereoselective aza-MRs. On the other hand, Enders et al. [22], Wang et al. [23] as well as Krishna et al. [24] highlighted the scope and catalytic performances of some organocatalysts in asymmetric aza-MRs. However, the last three review articles are almost 10 years old and they do not cover the application of many important organocatalysts, such as thioureas and nitrogen heterocyclic carbenes (NHCs) used for the asymmetric aza-MRs. Furthermore, in the last review article [24], the application of organocatalysts is included as a small part of a general review. In view of this, we considered it prudent to compile this mini review exclusively based on the application of all categories of the organocatalysts and highlighting their efficacies covering the literature of the last ten years.

## Review

In the present review, the known stereoselective syntheses of pharmacology-oriented nitrogen containing heterocyclic scaffolds via non-covalent bonding and covalent bonding organocatalytic aza-MRs has been systematized. This classification is especially useful for researchers to understand both the non-covalent and covalent organocatalysis.

It is intended to overview the literature of the last 10 years, i.e., from 2011 through 2020 only. Nevertheless, wherever necessary, earlier references may also be cited to maintain coherence. Furthermore, nitrogen nucleophiles comprise a large variety of compounds; however, in order to comply with the requirements of a mini review, additions of amines and amides only will be included.

### 1. Non-covalent bonding organocatalytic aza-Michael reactions

Organocatalysts catalyzing aza-MRs through mainly hydrogen bonding include cinchona alkaloids, squaramide derivatives, phase-transfer catalysts and bifunctional thiourea derivatives.

#### 1.1 Reactions catalyzed by chiral cinchona alkaloid derivatives

**Scheme 1:**
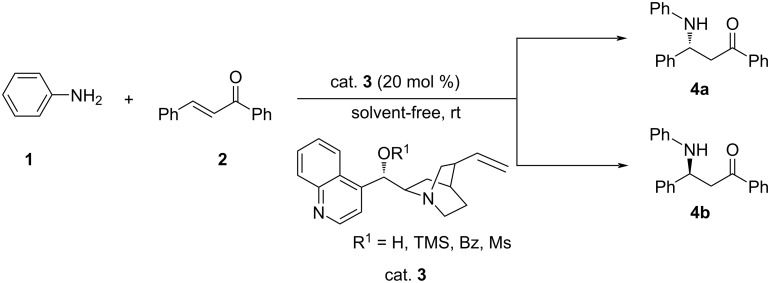
Asymmetric aza-Michael addition catalyzed by cinchona alkaloid derivatives.

Cai et al. prepared and used a number of organocatalysts from Cinchona alkaloids for the aza-MR of aniline (**1**) with chalcone (**2**) to obtain the adducts **4** in poor to very good yields (24 to >99%) with poor to moderate ee (9 to 55%). A complete reversal of stereoselectivity was observed on introducing a benzoyl group in cinchonine and cinchonidine. It was demonstrated that racemization occurred in suitable solvents under mild conditions due to retro-MR of the initially formed Michael adduct (Scheme 1) [25]. The proposed catalytic cycle involved generation of the active complex through hydrogen bonding between catalyst and aniline followed by interaction with chalcone via π–π stacking of aromatic rings and hydrogen bonding leading to the Michael adduct.

Likewise, Lee et al. reported cinchona-based primary amine catalyzed cascade aza-Michael-aldol reaction of α,β-unsaturated ketones **6** with 2-(1*H*-pyrrol-2-yl)-2-oxoacetates **5** where triphenylacetic acid was used as an additive. This cascade reaction afforded highly functionalized chiral pyrrolizines **8** in good yields (70–91%) with excellent levels of stereocontrol (≈92% ee*,* >20:1 dr in all cases). The ketone group in the cascade product was reduced asymmetrically to a chiral secondary hydroxy group (Table 1) [26].

**Table 1:** Asymmetric cascade aza-Michael−aldol reactions of α,β-unsaturated ketones with pyrroles.

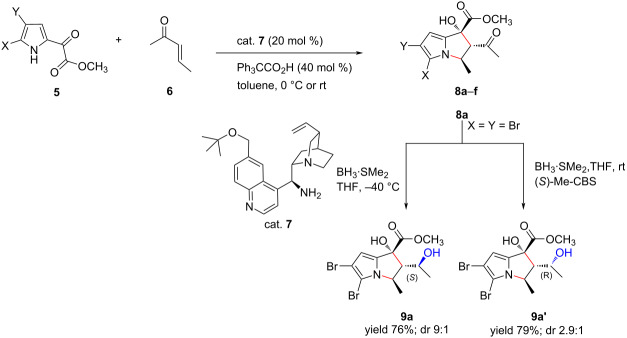					

**8**	X	Y	Yield [%]	ee [%]	dr

**a**	Br	Br	86	91	>20:1
**b**	Cl	Br	91	90	>20:1
**c**	I	Br	75	92	>20:1
**d**	Br	Cl	86	90	>20:1
**e**	Cl	I	73	90	>20:1
**f**	I	I	70	92	>20:1

In this case, the role of Ph_3_CCO_2_H as additive is to furnish the conjugate base Ph_3_CO_2_^−^ anion which subsequently deprotonates pyrrole to provide the stronger nucleophilic pyrrolide anion [27].

Similarly, Liu et al. accomplished an asymmetric intramolecular aza-Michael addition of various enone carbamates **10** using a chiral cinchona-based primary-tertiary diamine as catalyst to obtain 2-substituted piperidines **12** in good yields (75–95%) with up to 99% ee. Several sulfonic acids and carboxylic acids were tested as co-catalysts and trifluoroacetic acid (TFA) was found to give the best results [28]. Here the role of the co-catalyst is to assist in the formation of the iminium intermediate (Table 2) [29]. It appears that in this case, both activation mechanisms, namely through hydrogen bonding and iminium ion formation are operating.

**Table 2:** Intramolecular aza-Michael addition of conjugated ketones.

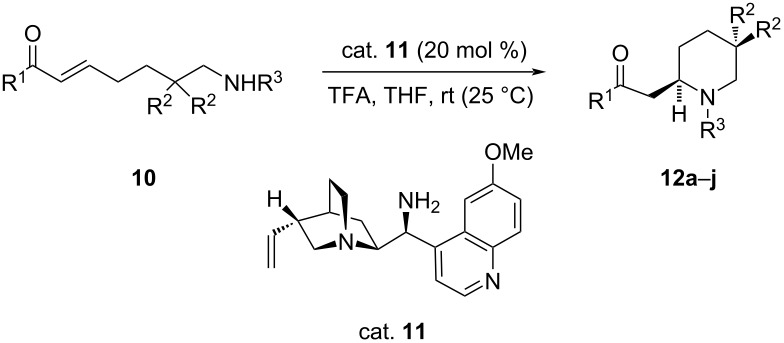					

**12**	R^1^	R^2^	R^3^	Yield [%]	ee [%]

**a**	Me	H	Cbz	95	98 (*R*)
**b**	Me	H	Boc	94	90
**c**	Me	Me	Cbz	97	99
**d**	Et	H	Cbz	94	96 (*R*)
**e**	iBu	H	Cbz	96	99
**f**	*n*-pentyl	H	Cbz	93	96 (*R*)
**g**	Ph	H	Cbz	95	96
**h**	4-Me-C_6_H_4_	H	Cbz	75	96
**i**	4-MeO-C_6_H_4_	H	Cbz	trace^a^	ND^b^
**j**	4-O_2_N-C_6_H_4_	H	Cbz	80	85

^a^The starting material was mainly recovered. ^b^ND = not determined.

Using the same chiral cinchona-based primary-tertiary diamine as catalyst (cat. **11**), Zhai et al. developed a highly efficient intramolecular enantioselective aza-Michael addition of carbamates, sulfonamides and acetamides **13** bearing an α,β-unsaturated ketone to synthesize a series of 2-substituted five- and six-membered heterocycles in good yields (up to 99%) and excellent enantioselectivity (92–97.5% ee) (Table 3). As in an earlier case [29], several acids were tested as co-catalysts and trifluoroacetic acid and diphenyl hydrogenphosphate (DPP) were found to give the best results [30].

**Table 3:** Intramolecular enantioselective aza-Michael addition.

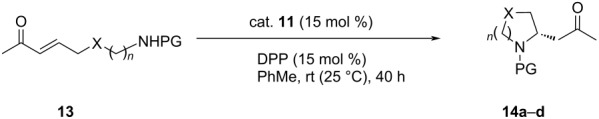					

**14**	X	*n*	PG	Yield [%]	ee [%]^a^

**a**	O	2	Boc	97	97
**b**	O	1	Boc	96	94
**c** ^b^	S	2	Cbz	55	95
**d**	S	1	Cbz	91	92

^a^Determined by means of chiral-phase HPLC analysis. ^b^Reaction time = 4 days.

**Scheme 2:**
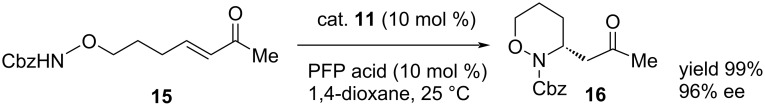
Intramolecular 6-*exo*-*trig* aza-Michael addition reaction.

Cheng et al. reported an intramolecular 6-*exo*-*trig* aza-MR of hydroxylamine-derived enone **15** for the synthesis of chiral 3-substituted 1,2-oxazinanes **16**. The catalyst **11** was used in this case also and pentafluoropropionic acid (PFP) was used as a co-catalyst. In the presence of 1,4-dioxane solvent, products chiral 3-substituted 1,2-oxazinanes (**16**) were obtained in 99% yield with good ee of 96% (Scheme 2) [31].

Following a similar strategy, Ma et al. accomplished a highly enantioselective aza-Michael addition of 4-nitrophthalimide (**17**) with α,β-unsaturated ketones **18** using 9-*epi*-9-amino-9-deoxyquinine **19** as the catalyst, the corresponding Michael adducts being obtained in moderate to good yields (49–98%) with excellent ee (95–99%) (Table 4) [32].

**Table 4:** Asymmetric aza-Michael addition of 4-nitrophthalimide to α,β-unsaturated ketones.

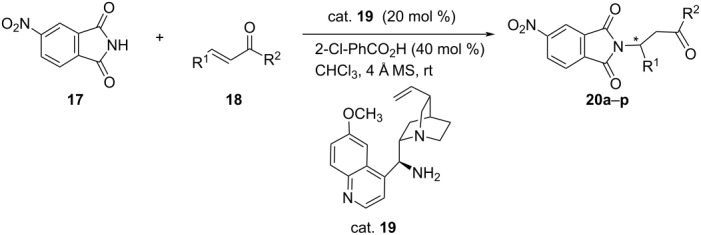				

**20**	R^1^	R^2^	Yield [%]	ee [%]

**a**	Ph	Ph	55	>99
**b**	2-Cl-C_6_H_4_	Ph	61	95
**c**	3-Cl-C_6_H_4_	Ph	65	98 (s)
**d**	4-Cl-C_6_H_4_	Ph	56	99
**e**	4-F-C_6_H_4_	Ph	60	>99
**f**	4-Br-C_6_H_4_	Ph	62	99
**g**	4-Me-C_6_H_4_	Ph	69	99
**h**	4-NO_2_-C_6_H_4_	Ph	49	>99
**i**	Ph	4-Cl-C_6_H_4_	54	99
**j**	4-F-C_6_H_4_	4-F-C_6_H_4_	71	99
**k**	iPr	Me	75	97
**l**	*n*-Pr	Me	88	96
**m**	*n-*Bu	Me	89	95
**n**	*n*-Pen	Me	98	95
**o**	*n*-Hex	Me	90	96
**p**	Me	Et	51	95

Jakkampudi et al. [33] adopted a different approach for the use of cinchona-based organocatalysts. Instead of using the cinchona derivative alone, they employed a mixture of cinchona derivative and amino acid such as ᴅ-proline, termed as the modularly designed organocatalyst (MDO) for the synthesis of bridged tetrahydroisoquinoline derivatives. It was perceived that the MDO self-assembled in situ from amino acids and cinchona alkaloid derivatives. For example, on reacting (*E*)-2-[2-(3-aryl-3-oxoprop-1-en-1-yl)phenyl]acetaldehydes **21** with ethyl or benzyl (*E*)-2-[(4-methoxyphenyl)imino]acetates **22** in the presence of the MDO **23/24** (quinidinethiourea + ᴅ-proline), instead of the expected domino Mannich/Michael product, the bridged tetrahydroisoquinoline product **25a** was obtained in high yield (90%) and excellent dr (94:6) and ee value (99%) (Table 5). The controlled reactions using **23** and **24** as the catalyst gave the product in very poor yield. It was concluded that the catalytic activity of the MDO was the result of the cooperative action of both constituents. Several examples of such MDOs are included in the paper. The reported yield varies from 56–90% with excellent ee ≈ 99% in all cases.

**Table 5:** Diastereoselective synthesis of bridged 1,2,3,4-tetrahydroisoquinoline derivatives using modularly designed organocatalyst.

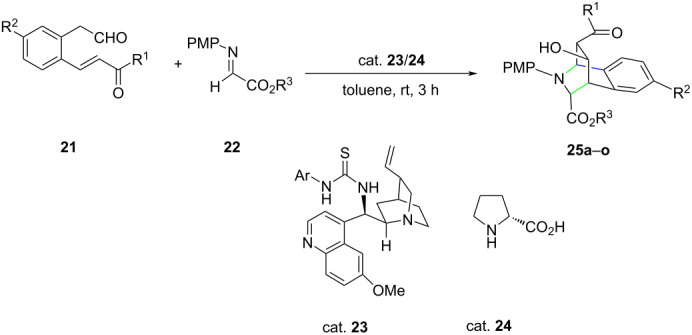						

**25**	R^1^	R^2^	R^3^	Yield [%]	ee [%]	dr

**a**	C_6_H_5_	H	Et	90	>99	94:6
**b**	4-F-C_6_H_4_	H	Et	81	>99	97:3
**c**	4-Cl-C_6_H_4_	H	Et	90	99	92:8
**d**	4-Br-C_6_H_4_	H	Et	80	>99	90:10
**e**	4-NC-C_6_H_4_	H	Et	77	99	87:13
**f**	4-Me-C_6_H_4_	H	Et	76	99	97:3
**g**	4-MeO-C_6_H_4_	H	Et	79	97	96:4
**h**	3-Cl-C_6_H_4_	H	Et	72	>99	88:12
**i** ^a^	2-F-C_6_H_4_	H	Et	–	–	–
**j** ^a^	2-Cl-C_6_H_4_	H	Et	–	–	–
**k**	C_6_H_5_	F	Et	73	>99	90:10
**l**	C_6_H_5_	MeO	Et	74	99	91:9
**m**	Me	H	Et	56	92	86:14
**n**	C_6_H_5_	H	Bn	75	99	89:11
**o**	4-Br-C_6_H_4_	H	Bn	77	>99	93:7

^a^Formation of a complex mixture was observed.

#### 1.2. Reactions catalyzed by chiral squaramide derivatives

Squaramides are related to cinchona alkaloids but are much more effective organocatalysts than the latter due to the ability of dual hydrogen bonding besides a tertiary nitrogen atom of quinuclidine nucleus which may serve both as an H-bond acceptor and a base in asymmetric Michael addition reactions [34–35].

In 2015, Zhao et al. synthesized spiro[pyrrolidine-3,3'-oxindoles] **29** in single step by asymmetric cascade aza-Michael/Michael addition reaction between 4-tosylaminobut-2-enoates **27** and 3-ylideneoxindoles **26** catalyzed by a chiral bifunctional tertiary amine, squaramide (cat. **28**) which afforded the corresponding adducts in good yields ranging from 72–99% with excellent diastereoselectivity (up to >99:1 dr) and enantioselectivity (>99% ee) (Table 6) [36].

**Table 6:** Synthesis of spiro[pyrrolidine-3,3'-oxindoles] via asymmetric cascade aza-Michael reaction catalyzed by squaramide.

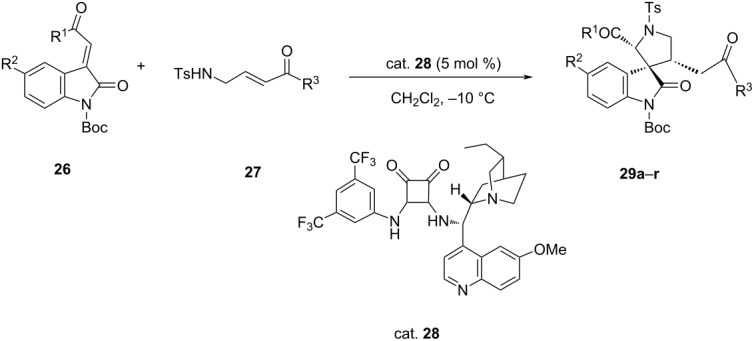						

**29**	R^1^	R^2^	R^3^	Yield [%]	ee [%]	dr

**a**	Ph	H	Me	72	98	96:4
**b**	Ph	H	O*t*-Bu	99	>99	93:7
**c**	Ph	H	OBn	99	>99	88:12
**d**	Ph	H	OEt	99	>99	65:35
**e**	4-FC_6_H_4_	H	O*t*-Bu	99	>99	88:12
**f**	4-ClC_6_H_4_	H	O*t*-Bu	99	>99	89:11
**g**	2-BrC_6_H_4_	H	O*t*-Bu	91	>99	85:15
**h**	4-BrC_6_H_4_	H	O*t*-Bu	99	>99	92:8
**i**	4-MeC_6_H_4_	H	O*t*-Bu	95	>99	96:4
**j**	3-MeOC_6_H_4_	H	O*t*-Bu	88	>99	92:8
**k**	4-MeOC_6_H_4_	H	O*t*-Bu	94	>99	89:11
**l**	2-naphthyl	H	O*t*-Bu	92	>99	89:11
**m**	2-thienyl	H	O*t*-Bu	96	>99	92:8
**n**	C_6_H_5_	F	O*t*-Bu	99	>99	89:11
**o**	C_6_H_5_	Cl	O*t*-Bu	99	>99	88:12
**p**	C_6_H_5_	Br	O*t*-Bu	99	>99	93:7
**q**	C_6_H_5_	Me	O*t*-Bu	99	>99	97:3
**r**	C_6_H_5_	OMe	O*t*-Bu	99	>99	99:1

In another report, Yang et al. accomplished a highly asymmetric cascade aza-Michael/Michael addition reaction for the synthesis of tetrahydroquinolines and tetrahydrochromanoquinolines catalyzed by a squaramide catalyst. The corresponding adducts were obtained in excellent yields with excellent diastereoselectivities and enantioselectivities (up to >99:1 dr, 99% ee) [37].

**Scheme 3:**
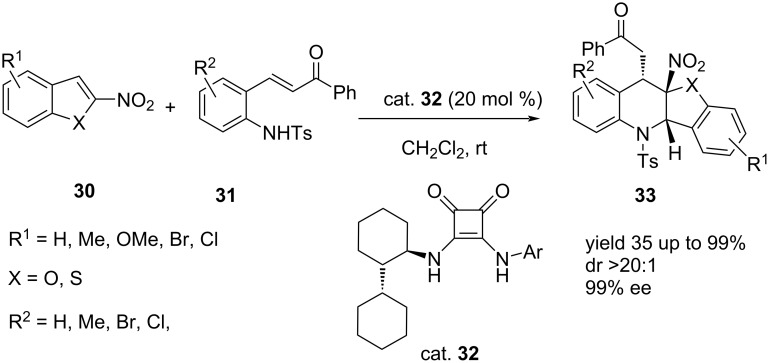
Asymmetric aza-Michael/Michael addition cascade reaction of 2-nitrobenzofurans and 2-nitrobenzothiophenes with 2-aminochalcones catalyzed by squaramide derivative.

Following a similar strategy, Zhou et al. obtained a series of optically active tetrahydrobenzofuro[3,2-*b*]quinolines and tetrahydrobenzo[4,5]thieno[3,2-*b*]quinolines **33** in high yields ranging from 35–99% and excellent diastereo- (>20:1 dr), and enantioselectivities (up to **≈**99% ee) (Scheme 3) [38].

Roy et al. accomplished an enantioselective intramolecular aza-Michael addition for the synthesis of dihydroisoquinoline and tetrahydropyridines from Michael reaction of *ortho*-homoformyl chalcone with various amines by using squaramide catalyst. The reaction occurred with good yields and excellent enantioselectivity [39].

Similarly, Li et al. reported an asymmetric cascade aza-Michael addition of 2-tosylaminoenones with unsaturated pyrazolones using squaramide as catalyst. The reaction proceeded smoothly under mild conditions to afford the corresponding spiro[pyrazolone-tetrahydroquinolines] in high yields (up to 99%) with excellent diastereoselectivities (up to >25:1 dr) and high enantioselectivities (up to 65–91%) [40].

Rajasekar et al. developed an efficient one-pot tandem rhodium(II)/chiral squaramide relay catalysis for the enantioselective construction of dihydro-β-carbolines **37** from the Michael reaction of suitably substituted indole derivatives **34** with *N*-sulfonyl-1,2,3-triazoles **35** in good yields (up to **≈**72%) and excellent enantioselectivity (up to 99% ee) (Table 7) [41].

**Table 7:** Asymmetric aza-Michael synthesis of dihydro-β-carbolines.

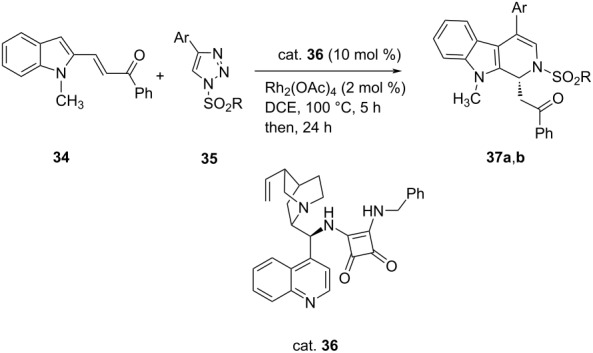				

**37**	Ar	R	Yield [%]	ee [%]

**a**	Ph	Ph	71	99
**b**	Ph	Me	72	80

In an interesting study, Wu et al. screened a number of cinchona derivatives and squaramides for their relative catalytic efficacies for the enantioselective aza-Michael additions between halogenated 2-hydroxypyridines (pyridin-2(1*H*)-ones) **38** and α,β-unsaturated 1,4-diketones or 1,4-ketoesters **39** in different solvents. The best results (yield 96%, ee >91%) were obtained on using squaramide catalyst in chloroform. However, for others, the yields ranged from 50–98% with good to excellent enantioselectivity (47–98% ee). The observed results were rationalized with density functional theory calculations (Table 8) [42].

**Table 8:** Asymmetric aza-Michael synthesis of *N*-substituted 2-pyridones.

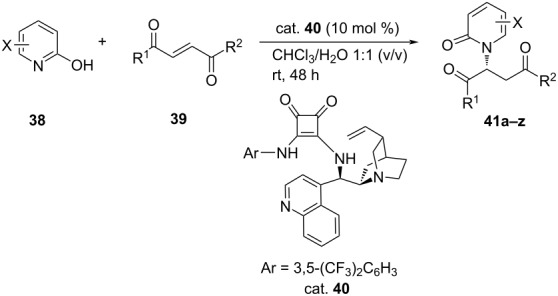					

**41**	X	R^1^	R^2^	Yield [%]	ee [%]

**a**	5-Br	Ph	Ph	98	98
**b**	5-I	Ph	Ph	78	91
**c**	5-F	Ph	Ph	60	74
**d**	H	Ph	Ph	50	44
**e**	3-Cl	Ph	Ph	88	93
**f**	3-Br	Ph	Ph	93	92
**g**	3-I	Ph	Ph	82	73
**h**	4-Br	Ph	Ph	60	47
**i**	6-Cl	Ph	Ph	0	–
**j**	5-Cl	*p-*F-C_6_H_4_	*p-*F-C_6_H_4_	93	93
**k**	5-Br	*p-*F-C_6_H_4_	*p-*F-C_6_H_4_	95	>99
**l**	5-I	*p-*F-C_6_H_4_	*p-*F-C_6_H_4_	87	97
**m**	3-Cl	*p-*F-C_6_H_4_	*p-*F-C_6_H_4_	82	99
**n**	3-Br	*p-*F-C_6_H_4_	*p-*F-C_6_H_4_	88	97
**o**	3-I	*p-*F-C_6_H_4_	*p-*F-C_6_H_4_	90	99
**p**	5-Cl	*p-*NC-C_6_H_4_	*p-*NC-C_6_H_4_	85	94
**q**	5-Br	*p-*NC-C_6_H_4_	*p-*NC-C_6_H_4_	73	97
**r**	5-Cl	*p-*Me-C_6_H_4_	*p-*Me-C_6_H_4_	70	35
**s**	5-Br	*p-*Me-C_6_H_4_	*p-*Me-C_6_H_4_	76	82
**t**	5-I	*p-*Me-C_6_H_4_	*p-*Me-C_6_H_4_	75	67
**u**	5-Cl	*p-*MeO-C_6_H_4_	*p-*MeO-C_6_H_4_	0	–
**v**	5-Cl	OEt	Ph	90	78
**w**	5-Br	OEt	Ph	82	80
**x**	5-F	OEt	Ph	83	63
**y**	3-Cl	OEt	Ph	70	90
**z**	3-Br	OEt	Ph	78	90
**aa**	4-Br	OEt	Ph	73	60
**ab**	3-Cl	OEt	*p-*F-C_6_H_4_	90	80

#### 1.3 Reactions catalyzed by chiral amines

He and co-workers developed heterogeneous synergistic catalysis using chiral amines SBA-15 (cat. **44**), which promote aza-Michael–Henry cascade reactions between 2-aminobenzaldehydes **42** and β-nitrostyrenes **43** to obtain chiral 3-nitro-1,2-dihydroquinolines **45** in good yields with up to 98% ee (Table 9) [43].

**Table 9:** Asymmetric aza-Michael–Henry cascade reaction.

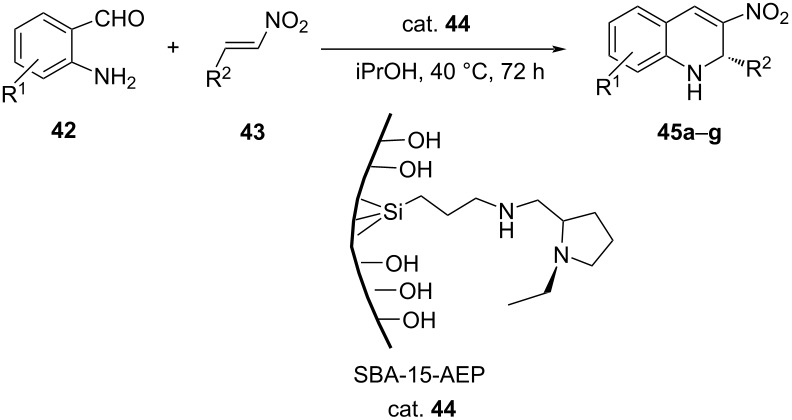				

**45**	R^1^ in **42**	R^2^	Yield [%]^a^	ee [%]^b^

**a**	H	2,3-(MeO)_2_-C_6_H_3_	67 (65)	98 (98)
**b**	H	4-Me-C_6_H_4_	60 (59)	90 (93)
**c**	H	2,4-(Cl)_2_-C_6_H_3_	45 (40)	97 (96)
**d**	H	3,4-(Cl)_2_-C_6_H_3_	42 (38)	95 (97)
**e**	3-MeO	Ph	52 (50)	95 (99)
**f**	5-Cl	Ph	55 (53)	98 (99)
**g**	3,5-Br_2_	Ph	68 (67)	99 (98)

^a^Determined by ^1^H NMR. ^b^Determined by HPLC. The data in parentheses are reproduced results.

#### 1.4 Reactions catalyzed by chiral phase-transfer catalysts

Chiral phase-transfer catalysts (PTC) have been recognized as versatile catalysts for the asymmetric aza-Michael addition reactions. Mahe et al. reported an effective, eco-friendly and cost-effective enantioselective synthesis of 3,5-diarylpyrazolines **49** by using phase-transfer methodology. They carried out a set of reactions between chalcones **46** and *N*-*tert*-butoxycarbonylhydrazine (**47**) in the presence of cesium carbonate and an *N*-benzylquininium salt as catalyst (cat. **48**) (solid–liquid phase-transfer conditions) to give the corresponding adducts in 40–90% yields with excellent ee of up to 99% (Table 10) [44].

**Table 10:** Asymmetric aza-Michael addition for the formation of (S)-(−)-pyrazoline.

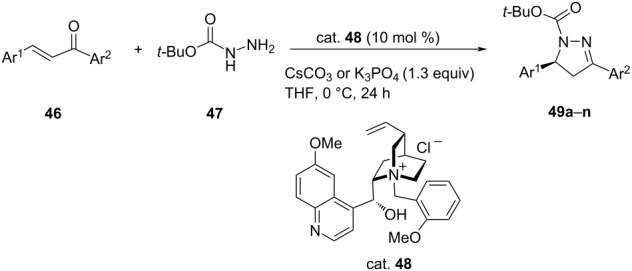				

**49**	Ar^1^	Ar^2^	Yield [%]	ee [%]

**a**	Ph	Ph	77	92 (−)
**b**	Ph	4*-*MeOC_6_H_4_	71	90 (−)
**c**	Ph	4*-*FC_6_H_4_	72	90 (−)
**d**	Ph	4*-*FC_6_H_4_	62	92 (−)
**e**	Ph	2*-*MeOC_6_H_4_	89	92 (−)
**f**	Ph	2*-*MeOC_6_H_4_	52	94 (−)
**g**	Ph	2-thienyl	66	87 (−)
**h**	Ph	2-thienyl	60	91 (−)
**i**	Ph	3,4*-*(Cl)_2_C_6_H_3_	40 (62)^a^	92 (−)
**j**	4*-*MeOC_6_H_4_	Ph	60	89 (+)
**k**	4*-*ClC_6_H_4_	Ph	70	88 (−)
**l**	2*-*MeC_6_H_4_	Ph	62	89 (−)
**m**	3-MeOC_6_H_4_	Ph	61	91 (−)
**n**	2-thienyl	Ph	46	78 (−)

^a^Yield determined by NMR analysis of the crude reaction mixture using an internal standard.

A different type of asymmetric aza-Michael addition was developed by Wang et al. They carried out asymmetric conjugate amination of *tert-*butylbenzyloxycarbamate (**50**) to β-nitrostyrene **51** under neutral phase-transfer conditions in the presence of chiral bifunctional tetraalkylammonium bromide (cat. **52**) in water-rich biphasic solvent. The reaction proceeded with high ee values of up to 95% and very good yields (**≈**99%) in all cases (Table 11) [45].

**Table 11:** Asymmetric aza-Michael addition reaction catalyzed by phase-transfer catalyst.

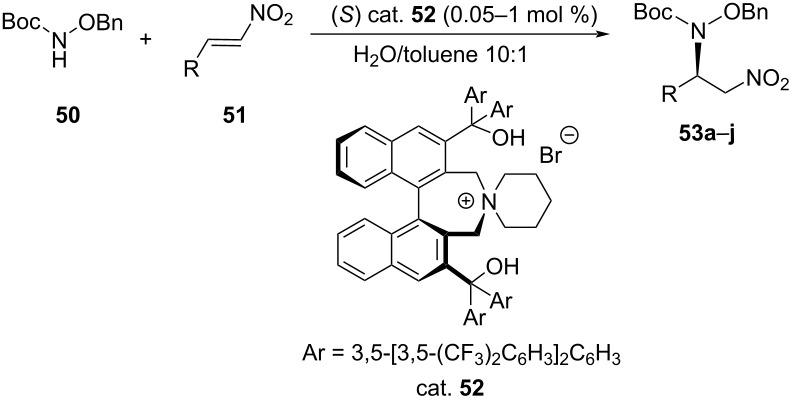							

**53**	Conditions^a^		R	Yield (%)		ee [%]	
		
	A	B		A	B	A	B

**a**	A	B	Ph	91	90	90	93
**b**	A	B	4-Me-C_6_H_4_	93	90	90	91
**c**	A	B	4-BrC_6_H_4_	91	89	91	94
**d**	A	B	3-FC_6_H_4_	70	62	90	93
**e**	A	B	4-TBSOC_6_H_4_	94	92	92	95
**f**	A	B	2-naphthyl	85	70	90	91
**g**	A	B	2-thienyl	93	81	90	94
**h**	A	B	2-furyl	90	82	90	93
**i**	A	B	(CH_3_)_2_CHCH_2_	95	99	77	82
**j**	A	B	*t*-Bu	97	99	79	83

^a^Conditions A: cat. (0.05 mol %) at rt or 0 °C, conditions B: cat. (1 mol %) at 0 °C.

Guo et al. synthesized a variety of benzoindolizidines (**56**) from α,β-unsaturated aminoketones **54** through intramolecular domino aza-Michael addition/alkylation reactions. The reactions were carried out in the presence of cinchona alkaloid-derived quaternary ammonium salts (cat. **55**) as the phase-transfer catalyst. The products were obtained in high yields (53–93%) with high enantioselectivities (40–76% ee) (Table 12) [46].

**Table 12:** Asymmetric aza-Michael/alkylation reaction catalyzed by cinchona alkaloid-derived quaternary ammonium salts.

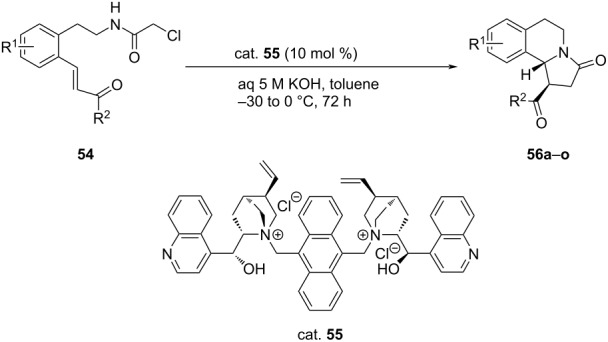				

**56**	R^1^	R^2^	Yield [%]	ee [%]

**a**	H	Ph	91	76
**b**	H	4-MeC_6_H_4_	84	69
**c**	H	4-MeOC_6_H_4_	53	65
**d**	H	Ph-C_6_H_4_	71	75
**e**	H	2-naphthyl	91	72
**f**	H	4-FC_6_H_4_	88	50
**g**	H	4-ClC_6_H_4_	93	80
**h**	H	4-BrC_6_H_4_	93	65
**i**	H	2-furyl	87	54
**j**	H	2-thienyl	91	68
**k**	H	2-Py	89	63
**l**	2-Br	Ph	86	85
**m**	2-NO_2_	Ph	75	85
**n**	3-MeO	Ph	87	40
**o**	H	Me	–	–

Lebrun et al. developed a new method to synthesize optically active isoindolinones via asymmetric intramolecular aza-MR by using phase-transfer catalysts. Alkenylated benzamide was used as the substrate in this reaction. The resulting compounds were found to be useful intermediates for the synthesis and development of benzodiazepine-receptor agonists [47].

In 2018, Sallio et al. worked on the same reaction by using different PTCs in order to improve yield and diastereomeric excess. They incorporated PTC and chiral auxiliary and reacted a variety of chiral phthalimidines **57** to obtain isoindolinones **59** in good yields (**≈**85%) with excellent de ranging 48–96% (Table 13) [48].

**Table 13:** Asymmetric aza-Michael synthesis of isoindolinones.

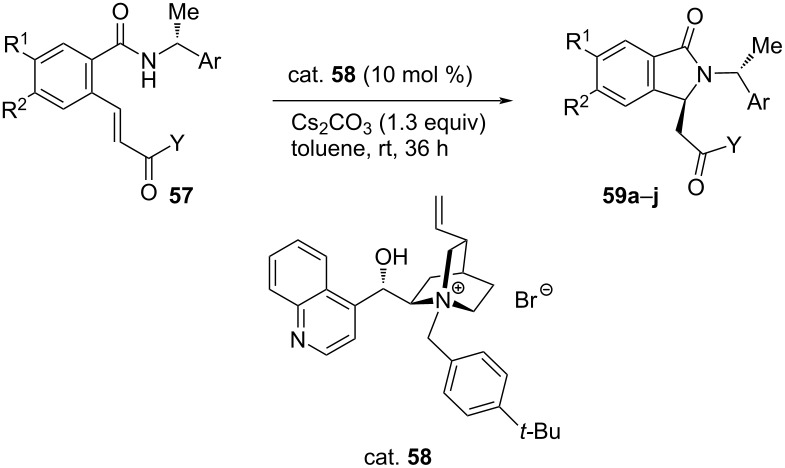						

**59**	R^1^	R^2^	Ar	Y	Yield [%]	de [%]

**a**	H	H	Ph	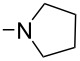	75	>96
**b**	H	H	Ph	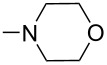	78	>96
**c**	H	H	Ph	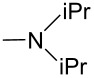	79	56
**d**	H	H	Ph	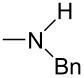	80	44
**e**	H	H	PMP^a^	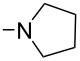	82	82
**f**	H	H	PMP	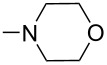	80	98
**g**	H	H	PMP	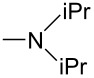	85	98
**h**	H	H	PMP	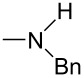	83	48
**i**	H	H	PMP	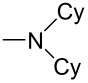	79	67
**j**	MeO	MeO	PMP	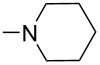	78	98

^a^PMP = *p*-methoxyphenyl.

#### 1.5 Catalysis by chiral bifunctional thioureas

Thioureas constitute one of the most important class of organocatalysts [49].

Wang et al. reported a cascade aza-Michael/Michael reaction of anilines **60** to nitroolefin enoates **61** using chiral bifunctional thiourea as catalyst (cat. **62**). It provided a mild and efficient approach to the synthesis of three stereocentered polysubstituted chiral 4-aminobenzopyrans **63** in high yields (71–96%) with excellent stereoselectivities of up to >99% ee (Table 14) [50].

**Table 14:** Asymmetric aza-Michael addition reaction catalysed by chiral bifunctional thiourea.

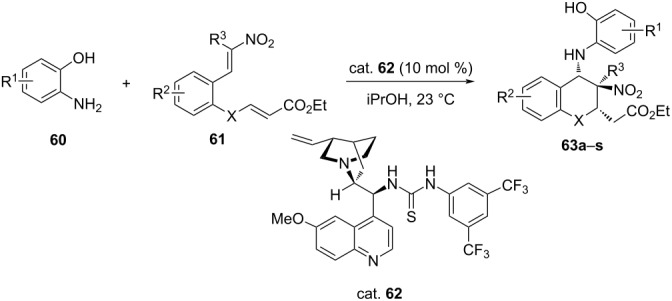							

**63**	R^1^	R^2^	R^3^	X	Yield [%]	ee [%]	dr

**a**	H	H	Me	O	96	96	>95:5
**b**	H	4-F	Me	O	71	>99	>95:5
**c**	H	4-Cl	Me	O	92	94	>95:5
**d**	H	4-Br	Me	O	84	94	>95:5
**e**	H	4-Me	Me	O	92	94	>95:5
**f**	H	4-MeO	Me	O	94	94	95:5
**g**	4-Me	5-MeO	Me	O	83	96	>95:5
**h**	4-Me	4-Br	Me	O	82	94	>95:5
**i**	5-Me	4-Br	Me	O	85	93	>95:5
**j**	6-Me	H	Me	O	94	93	>95:5
**k**	6-Me	5-MeO	Me	O	81	94	>95:5
**l**	4-Me	H	Me	O	94	96	>95:5
**m**	4-Br	H	Me	O	94	>99	>95:5
**n**	4-Cl	H	Me	O	89	93	>95:5
**o**	4-*t*-Bu	H	Me	O	91	94	>95:5
**p**	H	H	Et	O	91	96	>95:5
**q**	H	H	Bn	O	89	93	>95:5
**r**	H	H	Me	S	95	91	65:35
**s**	H	H	Me	S	93	94	95:5

In an interesting report, five organocatalysts belonging to three categories, namely cinchona alkaloid bases, bifunctional squaramides and thioureas were screened for the enantioselective *N*-alkylation of isoxazolin-5-ones via a 1,6-aza-Michael addition of isoxazolin-5-ones **64** to *p*-quinone methides (*p*-QMs) **65** to give isoxazolin-5-ones **67** bearing a chiral diarylmethyl moiety attached to the N atom. The best result in terms of enantioselectivity (85% ee) was obtained with quinine-derived thiourea in dichloroethane as the solvent. The scope of the reaction was also investigated *vis-a-vis* the effect of the substitution on the isoxazolinone ring and *p*-quinone methide (*p*-QM) partner. (Table 15) [51].

**Table 15:** Enantioselective 1,6-aza-Michael addition of isoxazolin-5-ones to *p-*quinone methides.

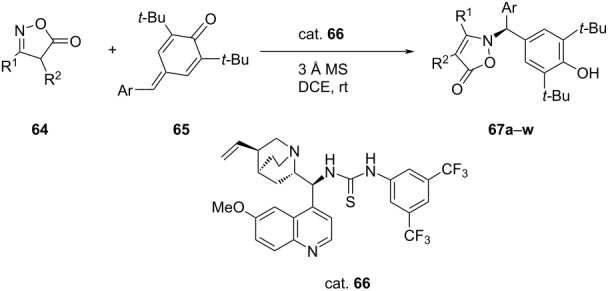					

**67**	R^1^	R^2^	Ar	Yield [%]	ee [%]^a^

**a**	Me	H	Ph	65	87
**b**	Et	H	Ph	51	81
**c**	Pr	H	Ph	50	81
**d**	Ph	H	Ph	77	54
**e**	Pr	H	Ph	78	89
**f**	Me	Me	Ph	66	62
**g**	Me	H	*p*-MeC_6_H_4_	62	88
**h**	Me	H	*p*-MeOC_6_H_4_	74	84
**i**	Me	H	*p*-ClC_6_H_4_	43	48
**j**	Me	H	*p*-O_2_NC_6_H_4_	47	89
**k**	Me	H	*o*-MeOC_6_H_4_	81	94
**l**	Me	H	*o*-ClC_6_H_4_	94	96
**m**	Me	H	*o*-BrC_6_H_4_	43	90
**n**	Me	H	*m*-MeOC_6_H_4_	20	25
**o**	Me	H	*m*-ClC_6_H_4_	36	81
**p**	Me	H	*m*-O_2_NC_6_H_4_	56	77
**q**	Pr	H	*p*-MeOC_6_H_4_	75	79
**r**	Pr	H	*p*-ClC_6_H_4_	78	88
**s**	Pr	H	*p*-O_2_NC_6_H_4_	80	86
**t**	Pr	H	*o*-ClC_6_H_4_	76	92
**u**	Pr	H	*m*-MeOC_6_H_4_	82	82
**v**	Pr	H	*m*-ClC_6_H_4_	80	88
**w**	Pr	H	Ph	71	86

^a^Determined by HPLC using a chiral stationary phase.

Takemoto and co-workers investigated three catalytic systems, namely arylboronic acid alone, its dual combination with chiral thiourea and integrated catalyst having boronic acid functionality in the chiral thiourea molecule. The dual combination of arylboronic acid with chiral thiourea was found as effective as arylboronic acid alone for the intermolecular asymmetric Michael addition of alk-2-enoic acids **68** with *O*-benzylhydroxylamine (**69**) giving racemic mixture of the product in poor yield. However, the integrated catalyst having boronic acid functionality in the chiral thiourea molecule gave the desired β-benzyloxyamino acid as the single product in a satisfactory yield. Thus, a series of these catalysts was screened. The best results in term of the yield (83%) and ee (90%) were obtained while using the catalyst having a *p*-nitrophenyl group on the other side of thiourea moiety in CCl_4_ in the presence of 4 Å molecular sieves (Table 16). The yields ranged 57–89% with ee 70–97% [52].

**Table 16:** Asymmetric intermolecular aza-Michael addition of (*E*)-3-substituted-2-enoic acid.

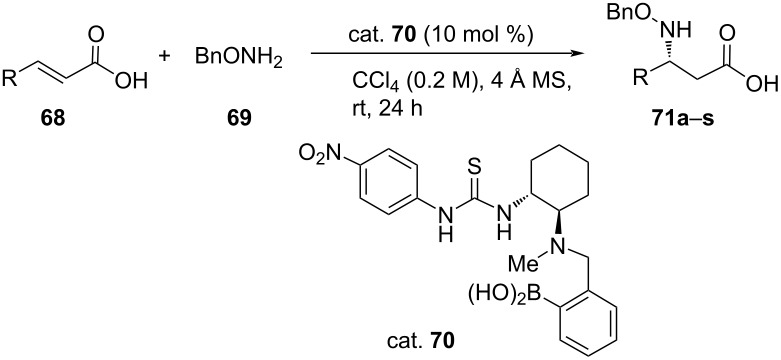			

**71**	R	Yield [%]	ee [%]

**a**	Me	75	97
**b**	Et	84	90
**c**	Pr	72	90
**d**	*n*-C_5_C_11_	76	88
**e**	*n*-C_7_C_15_	75	86
**f**	CH(CH_3_)_2_	57	76
**g**	CH_2_-OBn	89	85
**h**	(CH_2_)_3_-OBz	86	91
**i**	(CH_2_)_2_-SMe	78	87
**j**	(CH_2_)_3_-NHCbz	80	71
**k**	(CH_2_)_2_-C_6_H_4_-CF_3_-4	76	83
**l**	(CH_2_)_2_-C_6_H_4_-MeO-4	88	87
**m**	(CH_2_)_2_-C_6_H_4_-Br-2	85	88
**n**	(CH_2_)_2_-C_6_H_3_-3,4-(OMe)_2_	81	86
**o**	(CH_2_)_2_-2-naphthyl	90	87
**p**	(CH_2_)_3_-Ph	81	91
**q**	(CH_2_)_4_-Ph	84	88
**r**	CH_2_-Ph	80	71
**s**	Ph	0	–

A similar chiral multifunctional thiourea/boronic acid was used as an organocatalyst by Michigami et al. for the enantioselective synthesis of *N-*hydroxyaspartic acid derivatives **76** with perfect regioselectivity and high enantioselectivity (Table 17) [53].

**Table 17:** Asymmetric aza-Michael addition reaction for the synthesis of *N-*hydroxyaspartic acid derivatives catalyzed by chiral multifunctional thiourea/boronic acid.

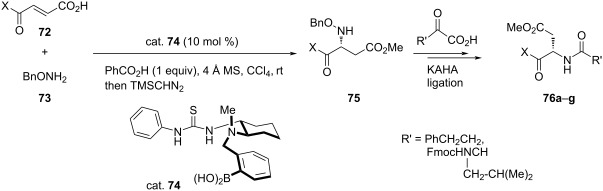				

**76**	X	Yield [%]	ee [%]	dr

**a**	*t-*BuO	88	93	–
**b**	BnO	50	91	–
**c**	EtO	49	94	–
**d**	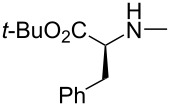	40	–	67:33
**e**	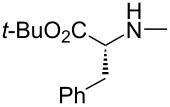	60	–	75:25
**f**	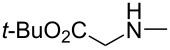	66	63	–
**g**	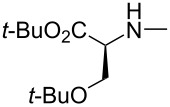	81	85	73:37

Likewise, Miyaji et al. reported an efficient method for the synthesis of 2-substituted indolines **79** via intramolecular aza-Michael addition of α,β-unsaturated carboxylic acid derivatives **77** in the presence of bifunctional thiourea organocatalysts (cat. **78**) (Table 18). The product was obtained in moderate to good yield of 53–99% with an ee of 74–93% [54].

**Table 18:** Intramolecular aza-Michael addition catalyzed by bifunctional thiourea.

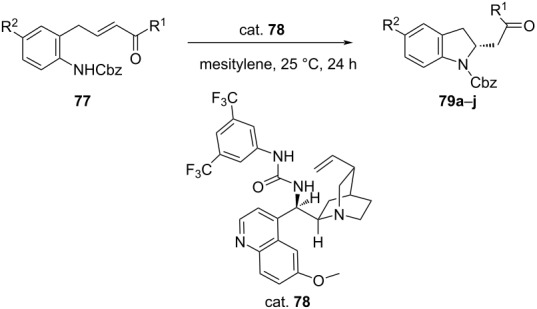				

**79**	R^1^	R^2^	Yield [%]	ee [%]

**a**	Ph	H	99	87
**b**	4-CH_3_OC_6_H_4_	H	73	84
**c**	4-CF_3_C_6_H_4_	H	79	88
**d**	2-naphthyl	H	83	88
**e**	4-BrC_6_H_4_	H	75	91
**f**	Ph	CH_3_O	82	83
**g**	Ph	F	69	82
**h**	Ph	Cl	82	84
**i**	4-BrC_6_H_4_	CH_3_O	53	93
**j**	CH_3_	H	18	74

Liu et al. accomplished a catalytic cascade aza-Michael–Henry-dehydration protocol for the preparation of chiral 3-nitro-1,2-dihydroquinolines **83** from the reaction of *N*-protected aminobenzaldehydes **80** with substituted nitroolefins **81** by using tertiary amine-thiourea catalyst (cat. **82**). This cascade reaction afforded aza-Michael adducts in 77–92% yields with high ee (up to 90%) (Table 19) [55].

**Table 19:** Intramolecular aza-Michael addition reaction catalyzed by tertiary amine-thiourea.

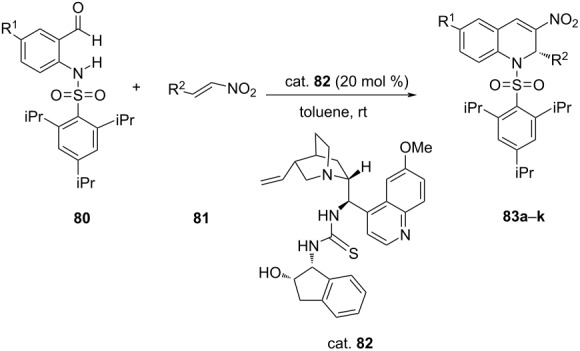				

**83**	R^1^	R^2^	Yield [%]	ee [%]

**a**	H	Ph	81	90
**b**	H	4-FC_6_H_4_	77	82
**c**	H	4-BrC_6_H_4_	91	85
**d**	H	4-NCC_6_H_4_	92	87
**e**	H	4-MeC_6_H_4_	83	81
**f**	H	4-MeOC_6_H_4_	75	84
**g**	H	3-ClC_6_H_4_	90	87
**h**	H	3-BrC_6_H_4_	86	89
**i**	H	3-MeC_6_H_4_	83	81
**j**	H	CH(Me)_2_	86	70
**k**	Cl	Ph	78	88

**Scheme 4:**
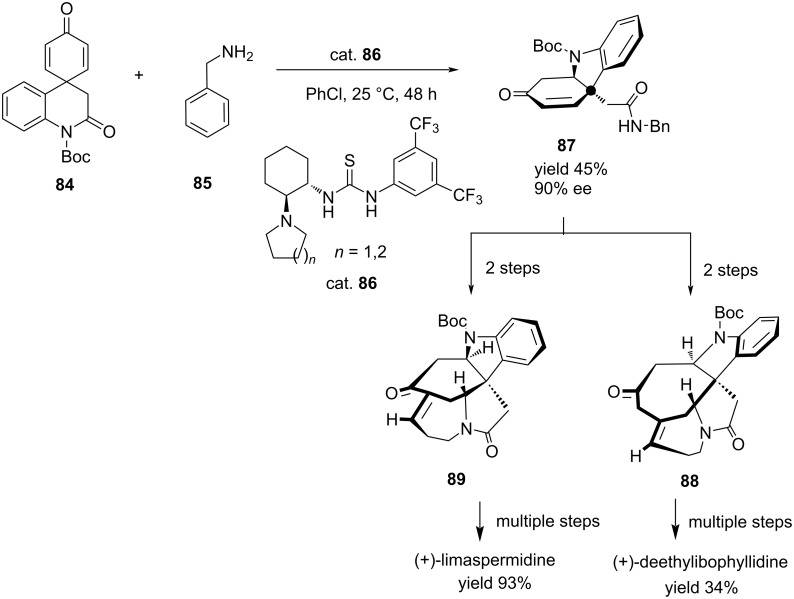
Asymmetric aza-Michael addition of *para*-dienone imide to benzylamine.

Du et al. developed an enantioselective catalytic tandem aminolysis/aza-Michael addition for the asymmetric total synthesis of two natural *Apocynaceae* alkaloids, (+)-deethylibophyllidine (**88**) and (+)-limaspermidine (**89**) from the reaction of *para*-dienone imide **84** with benzylamine (**85**) in the presence of bifunctional thiourea organocatalyst (Scheme 4) [56].

#### 1.6 Reactions catalyzed by chiral binol-derived phosphoric acids

Binol-derived chiral phosphoric acids have been shown to catalyze the reactions via single or double hydrogen bonding [57–58].

Saito et al. accomplished the chiral phosphoric acid-catalyzed intramolecular aza-Michael addition reaction of *N*-unprotected 2-aminophenyl vinyl ketones **90** to obtain chiral 2-substituted 2,3-dihydro-4-quinolones **92** in very good yields (67–95%) with high ee (82–97%) (Table 20) [59].

**Table 20:** Intramolecular aza-Michael addition reaction catalyzed by chiral phosphoric acid.

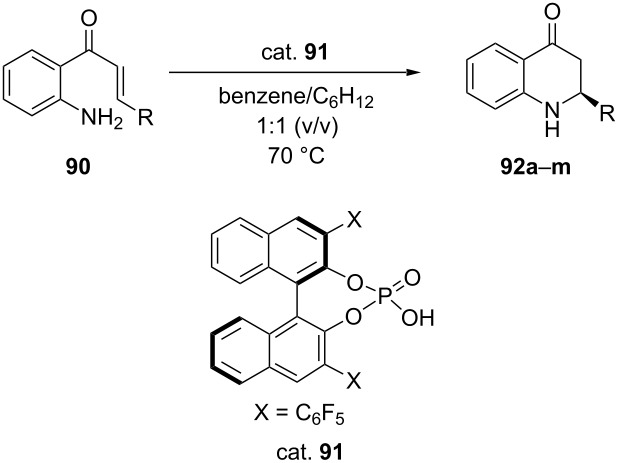			

**92**	R	Yield [%]	ee [%]

**a**	Ph	95	93
**b**	2-FC_6_H_4_	71	90
**c**	2-ClC_6_H_4_	90	93
**d**	2-BrC_6_H_4_	90	94
**e**	2-MeC_6_H_4_	97	88
**f**	3-BrC_6_H_4_	73	84
**g**	3-MeC_6_H_4_	95	86
**h**	3-MeOC_6_H_4_	95	92
**i**	4-FC_6_H_4_	quant	87
**j**	4-ClC_6_H_4_	67	82
**k**	4-MeC_6_H_4_	quant	97
**l**	2-naphthyl	82	81
**m**	*t*-Bu	64	88

Following a similar approach, Yang et al. reported asymmetric aza-Michael additions of anilines **94** to β-nitrostyrenes **93** using a chiral binol-derived phosphoric acid diester catalyst (cat. **95**). They succeeded in preparing β-nitroamines **96** in good yields (65–85%), but with only a moderate level of ee (19–70%) (Table 21) [60].

**Table 21:** Asymmetric aza-Michael addition of aniline to β-nitrostyrenes.

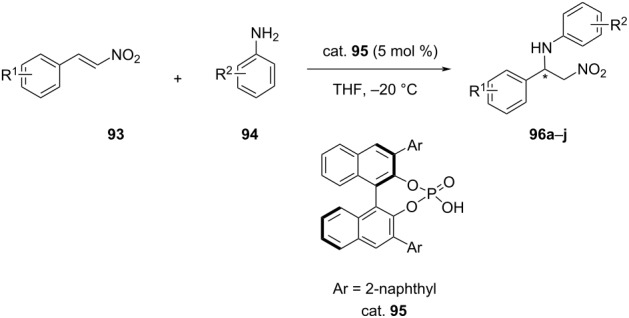				

**96**	R^1^	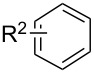	Yield [%]	ee [%]

**a**	4-Br	4-BrC_6_H_4_	82	19
**b**	4-Cl	4-BrC_6_H_4_	81	30
**c**	2-OMe	4-BrC_6_H_4_	65	44
**d**	4-OMe	4-BrC_6_H_4_	–	–
**e**	2-Br	4-BrC_6_H_4_	85	45
**f**	2,3-(OMe)_2_	4-BrC_6_H_4_	85	70
**g**	4-Me	Ph	70	30
**h**	4-Me	4-MeC_6_H_4_	75	30
**i**	4-Me	4-MeC_6_H_4_	70	42
**j**	4-Me	2-naphthyl	64	48

Feng et al. accomplished an asymmetric intramolecular aza-Michael addition of activated α,β-unsaturated ketones **97** by using chiral *N*-triflylphosphoramide as catalyst (cat. **98**). The products, namely 2-aryl-2,3-dihydro-4-quinolones **99** were obtained in good yields of up to 95% and good ee (58–72%) (Table 22) [61].

**Table 22:** Intramolecular aza-Michael addition reaction catalyzed by chiral *N*-triflylphosphoramide.

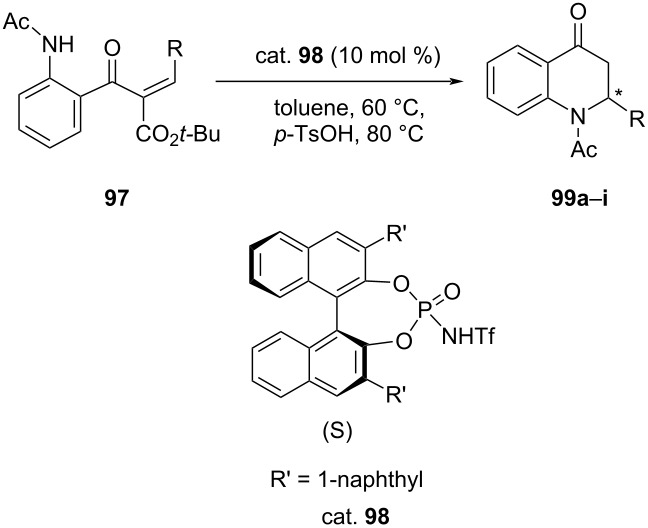			

**99**	R	Yield [%]	ee [%]

**a**	Ph	95	97 (−)
**b**	4-BrC_6_H_4_	90	58 (−)
**c**	4-ClC_6_H_4_	90	67 (−)
**d**	4-NO_2_C_6_H_4_	77	20 (−)
**e**	4-MeC_6_H_4_	98	82 (−)
**f**	4-MeOC_6_H_4_	94	60 (−)
**g**	2-MeOC_6_H_4_	95	4 (+)
**h**	1-naphthyl	81	76 (+)
**i**	2-naphthyl	98	76 (−)

### 2. Covalent-bonding organocatalysis of aza-Michael reactions

This category of organocatalysts includes N-heterocyclic carbenes and pyrrolidine derivatives.

#### 2.1 Catalysis by N-heterocyclic carbenes (NHC)

In recent years, NHCs have been used as organocatalysts for a wide variety of reactions [62].

Wang et al. investigated the use of several 1,2,4-triazolo-annelated chiral NHCs as organocatalysts to catalyze enantioselective aza-MR between primary amines (**100**) and β-trifluoromethyl-β-arylnitroolefins **101** and the best results (yield 99%, ee 91%) were obtained in the reaction of benzylamine (R^1^ = Ph) on using the NHC precursor as shown below in the presence of hexafluoroisopropanol (HFIP) as additive along with molecular sieves (4 Å) (Table 23) [63]. The role of HFIP is to act as proton shuttle, i.e., to assist in 1,3-prototropic shift.

**Table 23:** Aza-Michael addition of primary amines to β-trifluromethyl-β-phenylnitroolefin catalyzed nitrogen heterocyclic carbene.

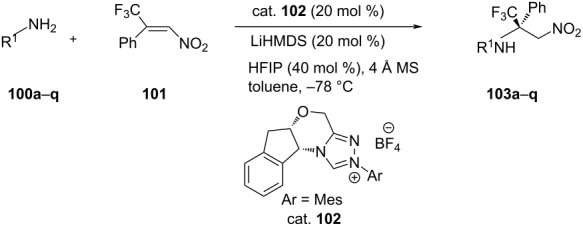			

**103**	R^1^	Yield [%]	ee [%]

**a**	C_6_H_5_CH_2_-	90	91
**b**	2-MeC_6_H_4_CH_2_-	67	91
**c**	4-MeOC_6_H_4_CH_2_-	89	92
**d**	3,5-(MeO)_2_C_6_H_3_CH_2_-	56	86
**e**	C_6_H_5_(CH_2_)_2_-	85	86
**f**	4-BrC_6_H_4_(CH_2_)_2_-	68	87
**g**	CH_3_(CH_2_)_2_-	80	92
**h**	C_7_H_15_CH_2_-	87	95
**i**	C_6_H_5_(CH_2_)_3_-	82	93
**j**	(CH_3_)_2_CH(CH_2_)_2_-	79	89
**k**	cyclopropyl-	79	97
**l**	cyclobutyl-	78	94
**m**	2-pyridylethyl-	87	93
**n**	BocHN(CH_2_)_2_	79	91
**o**	MeO(CH_2_)_3_-	87	87
**p**	(Me)_2_N(CH_2_)_2_-	99	91
**q**	2-thienyl(CH_2_)_2_-	98	93

#### 2.2 Catalysis by chiral pyrrolidine derivatives

Chiral pyrrolidine derivatives, such as (*S*)-proline are widely used as organocatalysts [54,64].

Lee et al. synthesized bromopyrrole alkaloids **107** via aza-Michael addition of 4,5-dibromo-1*H*-pyrrole-2-carbonitrile **104** to Bz-protected (*E*)-4-hydroxybut-2-enal **105** in the presence of (*S*)-α,α-bis[3,5-bis(trifluoromethyl)phenyl]-2-pyrrolidinemethanol trimethylsilyl ether as the organocatalyst (cat. **106**) and using PhCO_2_H as the acid additive. Desired products were obtained in good yields **≈**78% with excellent enantioselectivities of up to 93% (Table 24) [65]. The role of the additive is to assist in the formation of the iminium intermediate from the reaction of pyrrolidine with the aldehyde group.

**Table 24:** Asymmetric aza-Michael additions of pyrroles to protected (*E*)-4-hydroxybut-2-enals.

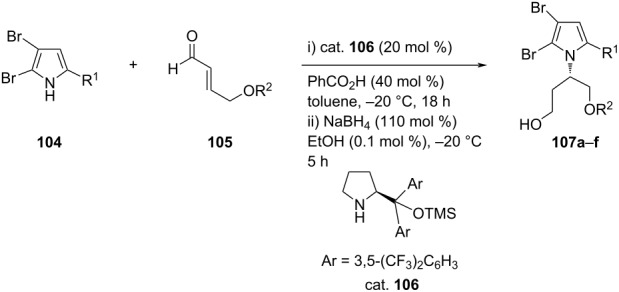				

**107**	R^1^	R^2^	Yield [%]	ee [%]

**a**	CN	Bz	70	76
**b**	CO_2_CH_3_	Bz	n.d.^a^	–
**c**	CN	TBS	78	80
**d**	CN	TBDPS	77	87
**e**	CN	TBDPS	76	91
**f**	CN	TBDPS	76	93

^a^Not determined.

Following a similar approach, Guo et al. accomplished the first organocatalytic asymmetric aza-Michael addition of purine bases **108** to aliphatic α,β-unsaturated aldehydes **109** and synthesized biologically active acyclonucleoside **110** via an iminium-ion activation mechanism. The initially formed product was reduced in situ to afford the final product in 82–89% yield and 89–96% ee (Table 25) [66].

**Table 25:** Asymmetric aza-Michael addition of purine bases to aliphatic α,β-unsaturated aldehydes.

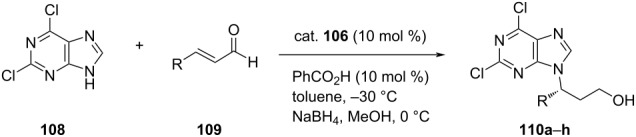			

**110**	R	Yield [%]	ee [%]

**a**	Me	87	89
**b**	Et	89	91
**c**	*n*-Pr	87	96
**d**	*n-*Bu	86	94
**e**	*n*-pentyl	85	96
**f**	*n*-hexyl	87	96
**g**	CH_2_OTBS	82	96
**h**	Ph	trace	–

In a similar method, Joie et al. accomplished an asymmetric organocatalytic quadruple cascade reaction of various α-ketoamides **111** with aromatic α,β-unsaturated aldehydes **112** to obtain tetraaryl-substituted 2-azabicyclo[3.3.0]octadienones **114** in good yields (34–71%) with excellent diastereo- and enantioselectivities (84–97%). The reaction occurred via an aza-Michael/aldol condensation/vinylogous Michael addition/aldol condensation sequence (Table 26) [67].

**Table 26:** Asymmetric aza-Michael organocatalytic quadruple cascade reaction.

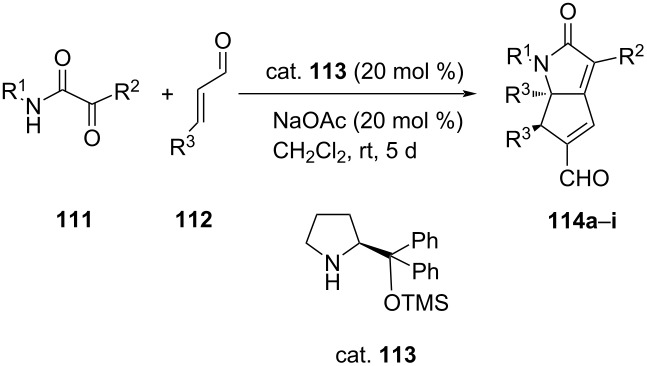					

**114**	R^1^	R^2^	R^3^	Yield [%]	ee [%]^a^

**a**	Ph	Ph	Ph	63	97
**b**	Ph	Ph	4-MeOC_6_H_4_	51	89 (91)
**c**	Ph	Ph	4-ClC_6_H_4_	34	85 (95)
**d**	Ph	Ph	2,3-(OCH_2_O)C_6_H_3_	56	84 (87)
**e**	4-MeOC_6_H_4_	Ph	Ph	66	92 (91)
**f**	3-ClC_6_H_4_	Ph	Ph	69	91 (95)
**g**	4-O_2_NC_6_H_4_	Ph	Ph	58	95
**h**	Ph	4-MeC_6_H_4_	Ph	70	88
**i**	Ph	4-ClC_6_H_4_	Ph	71	95

^a^Values in brackets correspond to the results obtained with the catalyst (*R*)-**113**.

Recently, the synthesis of axially chiral 4-naphthylquinoline-3-carbaldehydes **117** has been reported via Michael/Aldol cascade reaction of alkynals **116** with *N*-(2-(1-naphthoyl)phenyl)benzenesulfonamides **115** using the same pyrrolidine catalyst **113**. The products were obtained in excellent yields and enantioselectivities (Table 27) [68]. In this context, the presence of a strong electron-withdrawing sulfonyl group was found to be essential. On comparing efficacies of different sulfonyl groups, benzenesulfonyl moieties with electron-donating groups were found to be most effective. Furthermore, the utility of the newly developed method was demonstrated by preparing useful chiral 4-naphthylquinolines from the resulting products [68].

**Table 27:** Asymmetric synthesis of chiral 4-naphthylquinoline-3-carbaldehydes.

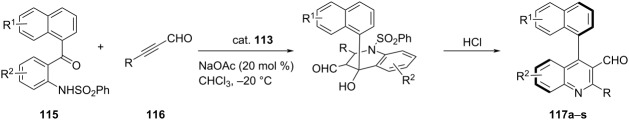					

**117**	R^1^	R^2^	R	Yield [%]	ee [%]

**a**	H	H	4-ClC_6_H_4_	97	93
**b**	H	H	Ph	94	95
**c**	H	H	4-FC_6_H_4_	86	94
**d**	H	H	4-BrC_6_H_4_	95	94
**e**	H	H	4-MeC_6_H_4_	81	94
**f**	H	H	4-MeOC_6_H_4_	86	95
**g**	H	H	3-ClC_6_H_4_	92	91
**h**	H	H	3-MeC_6_H_4_	88	93
**i**	H	H	3-MeOC_6_H_4_	83	93
**j**	H	H	*n*-C_5_H_11_	85	87
**k**	4-Me	H	4-ClC_6_H_4_	82	92
**l**	4-MeO	H	4-ClC_6_H_4_	91	90
**m**	4-F	H	4-ClC_6_H_4_	83	94
**n**	H	6-Cl	4-ClC_6_H_4_	90	94
**o**	H	7-Cl	4-ClC_6_H_4_	91	91
**p**	H	7-Cl	Ph	95	96
**q**	H	7-Cl	4-BrC_6_H_4_	95	94
**r**	H	7-Cl	3-MeC_6_H_4_	90	95
**s**	H	7-Cl	*n*-C_5_H_11_	88	90

**Scheme 5:**
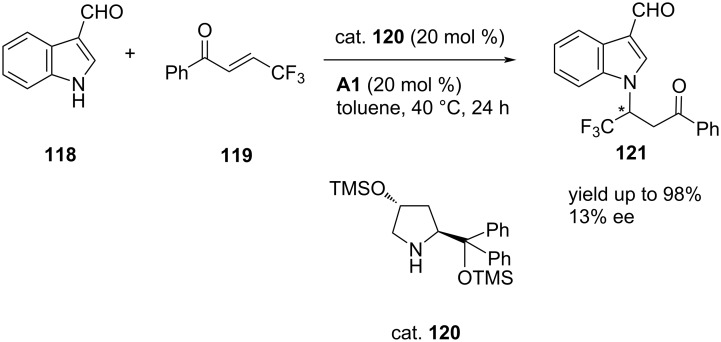
Asymmetric synthesis of chiral *N*-functionalized heteroarenes.

Chang-Jiang et al. developed a catalytic strategy by using a combination of prolinol silyl ether (cat. **120**) and benzoic acid (**A1**) catalysts to bring about reaction between 3-formyl-substituted indoles or pyrroles **118** and diverse electrophiles, including carbonyls, imines and other Michael acceptors (Scheme 5) [69]. The reaction with secondary amines occurred via the formation of HOMO raised dearomative aza-dienamine-type intermediates, which undergo direct aza-Michael addition to β-trifluoromethyl enones to afford *N*-functionalized heteroarenes **121** efficiently in moderate to excellent yields, albeit with low to fair enantioselectivity. However, asymmetric aza-Michael additions of these heteroarenes with crotonaldehyde yielded the adducts in moderate to good enantioselectivity under dual catalysis of chiral amines (Scheme 5) [69].

## Conclusion

The asymmetric aza-Michael reaction being a useful synthetic strategy for constructing C–N bonds to make a variety of nitrogen-containing chiral scaffolds of wide applications in the fields of pharmaceuticals, organic synthesis building blocks and accessible catalysis continues to attract attention of the chemists. During the last two decades, many new chiral organocatalysts have been developed for accomplishing these reactions with the nitrogen nucleophiles, such as aromatic amines and amides which are otherwise averse to reacting. The organocatalysts have emerged as catalysts of choice due to various reasons, such as their compatibility with the ‘Green Chemistry’ and possibility of tailoring them according to the requirements. Efforts are directed towards enhancing not only the yields of the products but also enantio- and diastereoselectivities of the aza-Michael reactions. New strategies have been adopted while making optimum utilization of the efficacies of the catalysts. Of these strategies, cascade reactions of the Michael addition in conjunction with one or more reactions leading to overall very high yields and ee are noteworthy. Another strategy of interest appears to be the generation of organocatalysts of enhanced efficacy in situ by mixing squaramides with amino acids again giving >99% ee. It may be perceived that in the coming years, more sophisticated methodologies will be developed with the advent of new organocatalysts to accomplish asymmetric aza-Michael reactions of even the so far unexplored and obstinate amines and amides substrates.
